# Feasibility of the music therapy assessment tool for awareness in disorders of consciousness (MATADOC) for use with pediatric populations

**DOI:** 10.3389/fpsyg.2015.00698

**Published:** 2015-05-27

**Authors:** Wendy L. Magee, Claire M. Ghetti, Alvin Moyer

**Affiliations:** ^1^Music Therapy Program, Boyer College of Music and Dance, Temple UniversityPhiladelphia, PA, USA; ^2^The Grieg Academy Music Therapy Research Centre, The Grieg Academy, University of BergenBergen, Norway; ^3^Elizabeth Seton Pediatric CenterYonkers, NY, USA

**Keywords:** pediatric, disorders of consciousness, brain injury, child, music therapy, assessment, measure, awareness

## Abstract

Measuring responsiveness to gain accurate diagnosis in populations with disorders of consciousness (DOC) is of central concern because these patients have such complex clinical presentations. Due to the uncertainty of accuracy for both behavioral and neurophysiological measures in DOC, combined assessment approaches are recommended. A number of standardized behavioral measures can be used with adults with DOC with minor to moderate reservations relating to the measures’ psychometric properties and clinical applicability. However, no measures have been standardized for use with pediatric DOC populations. When adapting adult measures for children, confounding factors include developmental considerations for language-based items included in all DOC measures. Given the lack of pediatric DOC measures, there is a pressing need for measures that are sensitive to the complex clinical presentations typical of DOC and that can accommodate the developmental levels of pediatric populations. The music therapy assessment tool for awareness in disorders of consciousness (MATADOC) is a music-based measure that has been standardized for adults with DOC. Given its emphasis on non-language based sensory stimuli, it is well-suited to pediatric populations spanning developmental stages. In a pre-pilot exploratory study, we examined the clinical utility of this measure and explored trends for test-retest and inter-rater agreement as well as its performance against external reference standards. In several cases, MATADOC items in the visual and auditory domains produced outcomes suggestive of higher level functioning when compared to outcomes provided by other DOC measures. Preliminary findings suggest that the MATADOC provides a useful protocol and measure for behavioral assessment and clinical treatment planning with pediatric DOC. Further research with a larger sample is warranted to test a version of the MATADOC that is refined to meet developmental needs of pediatric DOC populations.

## Introduction

Disorders of consciousness (DOC) refer to a compromised level of awareness of self and the environment, which manifests as coma, vegetative state (VS), or minimally conscious state (MCS). Consciousness is comprised of the dual dimensions of wakefulness and awareness (The Multi-Society Task Force on PVS, 1994), both of which are lacking in a coma state. Individuals who achieve wakefulness, but continue to lack awareness may be considered to be in a VS, “a clinical condition of complete unawareness of the self and the environment, accompanied by sleep–wake cycles with either complete or partial preservation of hypothalamic and brain-stem autonomic functions" (The Multi-Society Task Force on PVS, 1994, p. 1500). The nomenclature ‘VS’ remains contentious, and the European Task Force on DOC have made a proposal to replace this term with the term ‘unresponsive wakefulness syndrome’ (UWS: Laureys et al., 2010). However, as ‘UWS’ is not yet adopted internationally and is yet to receive recognition from authoritative sources (Royal College of Physicians, 2013), we use the term ‘VS’ in this paper. Individuals who emerge from a VS and demonstrate “minimal but definite evidence" of awareness of themselves or their environments may achieve a *MCS* (Giacino et al., 2002). Prolonged disorders of consciousness (PDOC) is a more recent term representing conditions of DOC that persist for more than 4 weeks following sudden acquired profound brain injury (Royal College of Physicians, 2013).

It is particularly challenging for clinicians to differentiate between VS and MCS. Despite the development and widespread dissemination of diagnostic criteria for VS and MCS, recent evidence of the rate of misdiagnosis of VS in adults (41%) corroborates previous estimates and suggests that a significant number of adults with consensus-based diagnoses of VS may actually be in MCS (Schnakers et al., 2009). Differential diagnosis is important for prognosis and treatment planning, including understanding an individual’s level of consciousness and potential for pain perception (Boly et al., 2008).

### Disorders of Consciousness in Pediatric Populations

Differential diagnosis may be particularly challenging in children, especially those who have developed DOC prior to the acquisition of foundational language and motor skills. Accurate worldwide estimates of the prevalence and incidence of VS and MCS in both adults and children are difficult to obtain due to variations in diagnostic criteria used within different geographic regions and across treatment settings (Pisa et al., 2014). Ashwal (2003, p. 537) estimates the prevalence of MCS in children under the age of 18 to be between 44 and 110 per 100,000 children, based on U.S. census data from 2000, when the overall population of children in the U.S. was 72,293,812. The estimated worldwide incidence of children (defined here as younger than 15 years of age) in a VS is approximately 93,000 (range 11,365–151,536), with an estimated 3,000 (range 367–4,897) children in a VS in the U.S. (Ashwal, 2004). Considerable variation exists between estimates of the prevalence and incidence of DOC in adults and children worldwide (Pisa et al., 2014), making the interpretation of these estimates challenging.

Children most commonly experience a DOC as a result of congenital or developmental disorders, acquired brain injuries (either traumatic or non-traumatic), or central nervous system degenerative and neurometabolic disorders (Ashwal, 2003). For example, in a group of 5,075 children diagnosed in VS or MCS, approximately 43% had an etiology of perinatal/genetic conditions, 15% had acquired brain injury, 2% had various degenerative diseases, and 40% were of unknown or unspecified etiology (Strauss et al., 2000). The specific presentation of DOC in children versus adults remains elusive, as there are very limited published data on the pediatric population demonstrating vegetative or MCS (Ashwal, 2005, 2013; Nicholas et al., 2014). Children with DOC may present differently than adults, especially when the DOC is acquired congenitally, and the child subsequently experiences developmental delays in all domains.

Diagnosis of DOC in children is challenging, particularly among those younger than 3 years of age, and those with significant developmental delays, due to immaturities in language and motor development. Lack of mature language and motor development confounds the assessment of cognitive function in this population, as such children are not able to complete tasks related to command following, verbal expression, or purposeful movement that are a part of existing assessment tools (Giacino et al., 2002). Without premorbidly developed language and motor skills, it becomes difficult to discriminate between children who are minimally conscious in a way that precludes motor ability, versus those who are fully conscious, but present as being minimally responsive due to compromised ability to execute motor or speech-based tasks required of assessments (Ashwal, 2003). Thus, the measures used for assessment and diagnosis of young children with DOC, or in children with significant developmental delays, must discriminate between minimal consciousness and minimal responsiveness.

Clinical guidelines established in the mid-1990s by the American Academy of Neurology and the Child Neurology Society defining aspects of the VS have largely retained their utility, though such guidelines may soon be updated due to recent advances in functional neuroimaging (Ashwal, 2013). Neuroimaging technologies have demonstrated that in isolated cases, some adults presenting as vegetative are able to activate areas of the brain in response to specific commands (Ashwal, 2013). Research exploring neurocognitive functioning in children has only recently commenced, and preliminary evidence of a single pediatric case report shows both consistencies and inconsistencies with brain imaging evidence from adults with DOC (Nicholas et al., 2014). At a minimum, children with DOC should receive “appropriate medical, nursing, or home care to maintain their personal dignity and hygiene” (Ashwal, 2013), but recent brain imaging evidence supports the need for more sophisticated forms of treatment and assessment for individuals with DOC. Evidence from neuroimaging may be particularly helpful in discriminating when a child is minimally conscious versus when he or she is minimally responsive (conscious, yet physically unable to complete physical or communicative acts; Ashwal, 2003).

Despite the challenges associated with diagnosis of DOC in pediatric populations, there are important clinical reasons for achieving accurate diagnosis. Accurately assessing the presence or absence of a particular disorder of consciousness and understanding typical trajectories of recovery serve as prerequisites for formulating appropriate treatment goals and providing family support and advisement for appropriate expectations (Ashwal, 2003; Pham et al., 2014). For example, in acute care and short-term rehabilitation settings, comprehensive interdisciplinary efforts can improve outcomes for children who experience a DOC as a result of an acquired TBI (Ashwal, 2003; Pham et al., 2014). The careful tracking of level of consciousness and responsivity over time can help demonstrate the impact of treatment. Children in MCSs, especially those meeting the criteria for “MCS+” (Bruno et al., 2011) have emerged to conscious states when given intensive multidisciplinary therapeutic intervention in the setting of acute inpatient rehabilitation (Pham et al., 2014). The relationship among diagnosis, prognosis, and treatment planning may take on different dimensions in long-term care settings, where children may receive comprehensive care services to promote quality of life and support families, regardless of a child’s particular DOC diagnosis. In palliative care settings, differential diagnosis may provide families with reassurance of the likely absence or presence of fluctuating awareness, which may provide insight as to what the child may be experiencing. Thus, the relevance of diagnosis in pediatric DOC may be partially dependent upon the care environment in which the child is situated.

### Measures of Awareness for DOC Populations

Establishing valid and reliable behavioral measures that are sensitive to the complex disabilities typical in DOC populations is of central concern due to the implications for treatment, care, and decisions around withdrawal of tube feeding (Seel et al., 2010). Although the Glasgow Coma Scale (GCS, Teasdale and Jennett, 1974) is a powerful predictor of mortality and morbidity in the acute phase (Zafonte et al., 1996), the behavioral domains tested in acute care and rehabilitation differ. In acute care the behaviors of interest are those concerning prognosis of survival, whereas rehabilitation is more concerned with diagnostic assessment, outcome prediction, projection of disposition needs, interdisciplinary treatment planning, and monitoring treatment effectiveness (Giacino et al., 2004). A number of DOC measures validated as diagnostic assessments assess responsiveness across the motor, auditory, and visual domains, measuring arousal and responses to verbal commands as well. Whilst each have different strengths, only the Coma Recovery Scale (Revised; Giacino and Kalmar, 2004) may be used with minor reservations to assess awareness in the person with DOC (Seel et al., 2010).

One of the problems with the existing behavioral measures of responsiveness for DOC is the reliance on language within assessment protocols. Following profound brain injury, receptive language is typically severely impaired which calls into question the value of language based assessments (Laureys and Schiff, 2012). This is further complicated by the high incidence of acquired impairments with the visual and/or motor domains that can limit a patient’s capacity to respond to verbal commands (Andrews et al., 1996). As the auditory modality has been found to be most sensitive in diagnosing awareness (Gill-Thwaites and Munday, 1999; Owen et al., 2005), attention has turned more recently to the importance of auditory stimuli in DOC assessments. Given its non-language basis and potential for emotional saliency, there is increasing interest in using music as a stimulus within DOC protocols for diagnosis (Okumura et al., 2014) and treatment (Verville et al., 2012; O’Kelly et al., 2013). The music therapy assessment tool for awareness in disorders of consciousness (MATADOC, Magee et al., 2014) is a measure used for assessment of awareness and intervention that uses a music-based protocol to stimulate responsiveness in DOC populations. A wide range of live musical stimuli is presented and behavioral responsiveness is rated across the motor, communication, arousal, visual, and auditory domains. Its Principal Subscale is reported to have good interrater reliability (mean = 0.83, SD = 0.11), good test–retest reliability (mean = 0.82, SD = 0.05), with good internal consistency (α = 0.76) with a strong first principal component (Magee et al., 2014). Rasch analysis confirmed it as a reliable, unidimensional and homogenous scale. Its performance against another validated sensory assessment as an external reference standard found excellent agreement (100%) for diagnostic outcomes of awareness states (Magee et al., 2014). The purpose of the MATADOC is to contribute to interdisciplinary clinical assessments of awareness in DOC patients by providing a rigorous and detailed assessment of auditory responsiveness. Although validated for use with adults, its value for use with a pediatric DOC population has not yet been tested.

### Pediatric DOC Measures

Valid pediatric specific DOC measures for accurate diagnosis of awareness and evaluation of treatment are lacking at the current time and there is no agreed gold standard for pediatrics (Cohen, 2009). Pediatric measures need to be developmentally appropriate, considering development milestones, such as the expectation to reliably follow verbal commands (Pham et al., 2014) or to localize painful stimuli (Durham et al., 2000). The existing rehabilitation measures for adult DOC cannot be adapted simply for children due to the reliance on language in their protocols. These require the child to have a fully developed use of language which is questionable when working with children who acquired brain damage prior to 5 years of age. Also, given that children most commonly experience a DOC as a result of congenital or developmental disorders rather than as a result of acquired conditions (Strauss et al., 2000), it is possible that youth with DOC will not have reached any developmental markers in the language domain. This highlights the complexity of developing appropriate measures for youth with DOC, and the need to develop measures that are not language-dependent. Thus, language specific items in adult measures compromise the validity of using such measures with children and require testing with children to meet the evidence-based criteria for clinical measures for DOC populations.

A number of behavioral coma measures have been developed to assist with nursing care of children in intensive care following catastrophic brain injury (Durham et al., 2000; Birse, 2006; Czaikowski et al., 2014), however, establishing reliability and validity for most of these measures remains outstanding (Cohen, 2009). Pediatric coma measures tend to have been developed for nursing staff to plan care and identify interprofessional collaboration where the child’s needs indicate it is required (Birse, 2006) although many require motor and verbal responses that are not appropriate for children under 2 years of age (Durham et al., 2000).

Some adult coma scales have been adapted for pediatric patients following recommendations that the verbal components of adult scales be modified when using these with children under the age of 4 years (American College of Surgeons, 1997, cited in Durham et al., 2000). For example, The Full Outline of UnResponsive Score Coma Scale (Wijdicks et al., 2005) was adapted in a pediatric version (the PFSS) by including age appropriate responses inclusive of all developmental milestones and age appropriate respiratory rates (Czaikowski et al., 2014). The Infant Face Scale (Durham et al., 2000) adapts elements of the adult GCS, basing its scoring on infant appropriate behaviors. Although reliability has been established to varying degrees for some of these measures (Durham et al., 2000; Birse, 2006; Cohen, 2009; Czaikowski et al., 2014), the existing pediatric coma scales are more suited to prediction of survival than prediction of functional outcome. There remains a need for rehabilitation measures that are valid for youth with DOC.

Sensory stimuli that promote purposeful responsiveness, without requiring previous acquisition of language, would seem to be indicated for assisting in the differential diagnosis of children with DOC. Misdiagnosing individuals as VS when they are in fact in MCS is most often a result of the inability to detect purposeful eye movement (Schnakers et al., 2009). However, due to the high incidence of cortical visual impairment observed in the pediatric DOC population (Huo et al., 1999; Hoyt, 2003), measurement tools that optimize the use of the auditory modality may increase the likelihood that clinicians can discriminate a child’s purposeful responses to sensory stimuli.

In human development, pre-linguistic communication is formed upon musical parameters such as pitch, dynamics, melodic contour, articulation, timing, and phrasing (Papousek, 1996; Trevarthen, 1999, 2002). In interactions with their environment, infants communicate immediate feeling states through varying these musical parameters, expressed through motor and vocal actions. The neurophysiological effects of music on children, either with brain damage or who are normally developing, is limited due to the practical and ethical complexities of researching this vulnerable population. However, there is some evidence that music can enhance neural processing of language mechanisms in at-risk children (Kraus et al., 2014). Brainstem assessments of children with Rett Syndrome have shown that music elicited responses comparable to normal neurophysiological responses suggesting that musical processing remains intact despite compromised neurological functioning (Bergström-Isacsson et al., 2014). Partially preserved brain activation patterns were found in response to salient auditory stimuli in one case of a child with PDOC (Nicholas et al., 2014), supporting the role for presenting stimuli with valence in the auditory modality. A model for using music with these children is therefore proposed given its role in normal child development, its neural effects with neurologically compromised populations, and the importance of optimizing environmental stimuli with personal saliency to enhance arousal in children with DOC (Amari et al., 2012).

Providing auditory stimuli in the form of musical sounds and interactions enables the clinician working with children with PDOC to provide a sound stimulus that is more intrinsically motivating than the noise stimuli frequently used in standardized neurobehavioral assessments of consciousness. The clinician can also make use of familiar and preferred sounds and music in order to promote the triggering of learned, cognitively mediated responses. Live musical stimuli may be presented with increasing complexity and stimulative qualities at a pace tolerated by the child with DOC, to enable the modulation to optimal arousal states for purposes of assessment. Thus, a standardized behavioral assessment tool for DOC that maximizes the use of musical stimuli, like the MATADOC, may promote the differential diagnosis of children with DOC.

Although the MATADOC is a standardized tool suitable for adult DOC populations, its utility with pediatric populations is not yet known. Understanding the importance of music in human development and its intrinsic motivating forces, it may similarly offer a valuable clinical measure for pediatric DOC. Its items need examining to determine their sensitivity to the developmental needs of pediatric populations and the appropriateness of the one language based item in particular. Following refinement of the measure, its reliability and validity need testing with a substantial pediatric sample. The purpose of this pre-pilot exploratory study was to examine the clinical utility of the MATADOC with a pediatric DOC cohort, explore trends for test–retest and inter-rater agreement, and compare the measure’s performance with that of external reference standards.

## Materials and Methods

### Recruitment

Children with PDOC were recruited from inpatient admissions to a pediatric long-term care facility providing specialized medical and therapeutic services to children with complex medical challenges. The pediatric skilled nursing facility provides residential care to children from birth to 21 years of age within a major metropolitan area. Participants between the ages of 2–18 years of age, who were assessed as having a DOC, were recruited from multidisciplinary treatment team referrals. Formal diagnoses of VS/MCS/Emerging had not been determined at the time of referral to the study. Children with known hearing impairments and profound visual impairments were excluded, as we wished to ensure that the clinical utility of these MATADOC items could be explored fully. Children with known musicogenic epilepsy were excluded due to the contra-indication of treatment, and children with seizure disorders that cause frequent and/or prolonged seizures were also excluded due to the complexity of behaviors and the risk of collecting skewed data.

This study involved only children who could not communicate using language, and potentially involved children who had acquired their brain injury before language skills were fully developed. As language development was a variable of specific interest in this study, only children whose first language was English were included in this pilot study. For the purposes of examining whether language dependent items impact upon the validity and reliability of the MATADOC, recruitment aimed to include 50% participants who had never developed language skills (i.e., those who had acquired a DOC prior to the age of 3 years) and 50% that had developed language skills prior to acquiring a DOC (i.e., those who had acquired a DOC from the age of five and older). Consent for involvement was gained from the children’s legal guardians. This study received ethical approval from the Behavioral and Social Sciences Committee of the Institutional Review Board at Temple University, Philadelphia, as well as from a research advisory committee at the clinical facility.

### Procedure

Each child received a MATADOC assessment that involved four individual clinical contacts. Clinical sessions were scheduled at a time of the day to suit the child’s usual schedule of school and treatment. The MATADOC protocol was implemented by a Board Certified Music Therapist who was experienced in work with children with DOC and had been trained to a recognized level of competency in delivering the MATADOC. The MATADOC protocol involves the use of live music in a process of at least five tasks that aim to determine the patient’s awareness of and responsiveness to musical stimuli. The tasks include musical entrainment to the child’s breathing whilst singing the child’s name; presenting a song known to be preferred or at least familiar to the child; and presenting musically related visual and auditory stimuli (Magee et al., 2012). If the child demonstrated responses during these tasks that indicated higher level responsiveness (e.g., attempts to vocalize to music; attempts to touch or play an instrument), the protocol’s tasks were adapted and extended in the moment to assess the child’s responsiveness within a particular domain of functional behavior.

In order to enhance comfort and promote familiarity with the setting, clinical interventions consistently took place in the child’s individual room or in a specific music therapy clinical room, depending upon which setting offered the most controlled and appropriate environment for the child. Distractions (e.g., interruptions; environmental noise) were minimized as much as possible. Children were physically positioned to enhance wakefulness and physical comfort. This was usually in their wheelchair or in their bed, following the recommendations of each child’s care team.

### Data Collection

#### Measures

The MATADOC has 14 items that measure responsiveness across auditory, visual, arousal, physical, cognitive, communication, and emotional behavioral domains. The MATADOC data collectors (assessors) were four Board Certified Music Therapists who were experienced at working with children with DOC (mean = 8.5 years; range = 5–12 years). They were all trained to a specified level of competence in delivering the MATADOC protocol and rating responses.

MATADOC data were collected for each child during four individual clinical contacts that took place over an 8 day period. Data were collected using the MATADOC rating form for each clinical contact by two assessors: one who delivered the protocol (therapist rater) and one who observed the clinical intervention (observer rater). Assessors remained blind to each other’s ratings. MATADOC clinical contacts were video recorded. This allowed for raters to perform a further rating at a later date from the video. In this way, we captured performance trends for test–retest ratings in addition to inter-rater ratings.

Selecting appropriate measures as external reference standards in this study was problematic given the lack of measures validated for pediatric DOC that are suitable for rehabilitation settings. With no “gold standard” measure, expert opinion and common clinical practice with pediatric DOC was sought by surveying two global networks specializing in neuro-rehabilitation. From the 11 measures that are reportedly used in pediatric acute and rehabilitation settings, two were selected considering existing evidence-based recommendations for adults (Seel et al., 2010), the relevance of each measure to assessing sensory responsiveness, measures used in previous studies of children with DOC and local practices in the facility at which this study took place. The Coma Recovery Scale (Revised; CRS-R, Giacino and Kalmar, 2004), the COMA/Near Coma Scale (CNC, Rappaport et al., 1992), and the Pediatric Center Criteria Persistent VS (described later) were used as external measures to compare with the MATADOC (Magee et al., 2014). All non-MATADOC data were collected by an attending physician at the facility who was experienced with children with DOC and was blind to MATADOC ratings.

The CRS-R is a measure used to estimate the incidence of selected neurobehavioral signs in patients admitted to rehabilitation with a diagnosis of DOC (Giacino et al., 2004). It has 25 items that are hierarchically arranged items comprising six subscales addressing auditory, visual, motor, oromotor, communication, and arousal processes. It was selected for this study as, although not validated for pediatric populations, it is recommended for use with adult DOC with only “minor reservations” (Seel et al., 2010). It has also been used in previous studies with children with DOC caused by acquired brain injury (Patrick et al., 2000; McMahon et al., 2009).

The Coma/Near Coma Scale was designed to measure subtle changes in responsiveness in individuals with severe brain injury and consists of eleven items assessing responses to auditory, visual, olfactory, tactile, pain, and reflexive stimuli as well as vocalizations and response to commands (Rappaport et al., 1992). The presented stimuli include a range of verbal commands, sounds, olfactory, and tactile stimuli including unpleasant stimuli. It has been found to have high inter-rater reliability and validity for use with a DOC population (Rappaport et al., 1992). Although not validated for use with pediatric populations, it has been used in previous studies with children with DOC caused by acquired brain injury (Patrick et al., 2000; McMahon et al., 2009; Pham et al., 2014).

The Pediatric Center Criteria for Diagnosing a Persistent Vegetative State (PCC) is a measure that was designed within the facility at which this study took place and was regularly used in routine clinical care. It is a checklist designed as a series of yes/no questions that would differentiate a persistent VS from a MCS. It was based on the definition of MCS proposed by the Aspen Neurobehavioral Conference Workgroup in 2002 (Giacino et al., 2002). Recognizing that most children in the facility were either congenitally affected, or acquired their brain injury prior to the acquisition of language skills, this checklist did not require any specific validated assessment tool that relied on language comprehension. Instead, an interdisciplinary team would reach a consensus based on subjective assessments of five criteria: presence of voluntary actions or behavior, voluntary language or expression, sustained eye tracking, cognitive responses, or specific responses to commands. If any one of the criteria were reliably observed by a member of the interdisciplinary team, then a diagnosis of MCS was made. The interdisciplinary team consisted of the physician and might include any number of the following: nursing, social work, physical therapy, occupational therapy, speech/language pathology, music therapy, art therapy, child life, and/or recreation therapy. Staff was educated by the physician regarding the nature of spinal, brainstem, and limbic reflexes as well as other non-volitional movements such as seizures and myoclonus.

Non-MATADOC data were collected in single assessments that occurred within the same time period as the MATADOC ratings. All assessors (MATADOC and non-MATADOC) remained blind to all other ratings.

## Results

Four children were recruited over a 3 month period from residents admitted to the facility for continuing care. We had hoped to recruit a further two participants, however, a number of potential participants were excluded due to a diagnosis of visual impairment and/or lack of English as a primary language. All the children had acquired brain damage sustained after birth, resulting in profound physical, cognitive, and communication impairments and were fully dependent for all activities of daily living. All the participants had been screened to have DOC but had not received diagnoses through formalized assessments, e.g., VS/MCS/Emerging. Data were collected using paper forms of the four study measures and then entered into an Excel spreadsheet by an independent research assistant, and double checked for accuracy by the principal investigator.

Data were analyzed by two of the investigators by means of descriptive statistics as the sample size precluded the use of inferential statistical analysis. Initially, we calculated the frequencies of each assessor’s ratings for all MATADOC items for the live condition and the video condition to examine trends within each child’s MATADOC results. Mean percentage agreement between raters was then calculated for combined conditions within each participant’s data. We then examined ratings from both assessors across the four participants’ ratings to look at the performance of each MATADOC item. To examine the overall performance of the MATADOC, mean percentages of agreement and disagreement in inter-rater and test–retest ratings were calculated. In inter-rater comparisons, we explored whether one of the assessors in the different roles (therapist rater vs. observer rater) rated responses higher or lower more consistently. We also paid particular attention to patterns of ratings made during live sessions versus video recorded sessions in order to make recommendations for the optimal design of further research with this measure. All MATADOC outcomes for each child were calculated by pooling the ratings from both live and video conditions of that child’s assessment. This was done in order to balance occasional discrepancies in outcomes between the live and video conditions, as we anticipated that live ratings might provide more favorable outcomes than ratings from the video condition. For both inter-rater and test–retest comparisons, we considered mean agreement in the upper quartile (75–100%) only as “good,” in line with rating schemes for agreement drawn from those used by the DOC task force (Seel et al., 2010). Data were analyzed by the first author (WM) and then checked by the second author (CG) for accuracy and assessment of the level of agreement. Where there was disagreement, the authors reviewed the data until agreement was reached.

### Comparisons of Items Across Measures

Patterns of diagnostic outcome across comparable items of the four measures were examined to explore divergence and similarities between the MATADOC items and more widely used measures. This comparison was undertaken to identify potential sensitivities or weaknesses in MATADOC items so as to assist with refining the MATADOC for further testing with a pediatric cohort.

All four measures test visual responsiveness, although the CNC has two items for this domain (labeled Visual items 1 and 2 in the tables). Only the MATADOC, CRS-R, and CNC test auditory responsiveness. A second item in the MATADOC called “Awareness of musical stimuli” also falls under the auditory domain, where raters record behaviors that “evidence... the patient’s awareness of the music” played in the patient’s environment (Magee et al., 2012). Thus the outcomes of this item are provided with the label of “Auditory item 2.” All four measures rate responses to verbal commands. The MATADOC, CRS-R, and PCC rate responsiveness in the motor domain. The MATADOC and CNC rate vocalization, the PCC rates “voluntary language or expression,” and the CRS-R rates “Oromotor/Verbal Function”: these items are compared with each other under the category “Expression.” The CNC does not rate this item when a tracheostomy is in place. Although all four measures rate arousal, these ratings are not associated with diagnosis and so have been omitted from the results reported here.

Because this study aimed to explore the clinical utility of the MATADOC and make recommendations for its refinement and future research, we examined inter-rater agreement for the MATADOC within each of the live and video conditions as well as overall agreement across conditions. A pattern of difference in rating emerged early in analysis between ratings made by each rater (i.e., one rating higher than the other consistently) and by condition. Therefore we examined these trends in detail. Test–retest agreement is also reported.

As we were interested in how the MATADOC performed with both children who had acquired language prior to DOC (*n* = 2) and those who had not (*n* = 2), we present the results in a case by case form as the results were widely divergent across the four participants. The scores of comparable items for all measures are presented in tables for each individual.

### Case 1

This 8-years-old female had sustained an anoxic brain injury from a cardio-pulmonary attack 2 years prior to data collection following normal development including normal language development (see **Table 1**). She demonstrated minimal responsiveness for all items across all four measures, resulting in a diagnosis of VS. Inter-rater agreement on MATADOC ratings was generally weak (66%), influenced by the therapist–rater rating responses during the live condition higher than those rated by the observer–rater 38% of times. This compared with the video condition where the therapist–rater ratings were higher than the observer–rater ’s only 13% of times. Inter-rater agreement was best for items that rated clearly observable behaviors (e.g., Arousal: 87.5%; Change in Eye Contact/Direction: 75%) and also for items that rated cognitively mediated behaviors that were plainly absent in such a minimally responsive patient (e.g., Verbal Command: 100%; Choicemaking: 100%). Overall, the level of agreement between and within raters was best when a behavior was rated as “absent” or “VS.” Of particular note is the disagreement between the two raters on the child’s sensory responsiveness in both live and video conditions, i.e., Visual responsiveness: Item 1; Auditory responsiveness: Item 2. Overall test–retest agreement was mixed (79%) due to the therapist–rater’s higher ratings during the live condition. There was better test–retest agreement for items rating behaviors that were unambiguously absent, with one exception for the item “Attention to task” where there was 87.5% test–retest agreement for responses categorized as “MCS.” The observer–rater’s ratings demonstrated excellent test–retest agreement (10 items at 100%; 5 items at 75%) although the child’s minimal responsiveness, resulting in many items rated as “0/VS,” assisted with this test–retest agreement.

**Table 1:** Participant 1: demographic information and results from four disorders of consciousness (DOC) measures.

Age	8 years			
Gender	F			
Diagnosis	Anoxic brain injury and encephalopathy. Seizure disorder. Spastic quadriparesis		
Time since injury	2 years			
Language prior to brain damage	Yes			
Average session duration	19.25 min			
Inter-rater agreement	66%			
Test–retest agreement	79%			
Gender	F			
	**Measures**			
	**MATADOC**	**CRS-R**	**PCC**	**CNC**

Diagnostic outcome	VS	VS	PVS	Marked coma^∗^
Visual item 1	VS	VS	VS	VS
Visual item 2	–	–	–	VS
Auditory item 1	VS	VS	–	VS
Auditory item 2	VS	–	–	–
Verbal command	VS	VS	VS	VS
Motor	VS	VS	VS	–
Expression	VS	VS	VS	NA^∗∗^

*^∗^Inconsistently responsive to stimulation presented to one sensory modality and not responsive to simple commands. No vocalization. ^∗∗^Not assessed due to tracheostomy in situ*.

### Case 2

An 8.5 years-old boy had been developing normally prior to acquiring profound brain damage from a viral infection, which occurred over 7 years prior to data collection (see **Table 2**). Given the age he acquired his brain damage (1.5 years of age), he was considered not to have full language development. Diagnostic outcomes provided by DOC measures were not in agreement: although the CRS-R, PCC, and CNC all gave a diagnosis of VS, this contrasted with the MATADOC outcome of MCS. A closer examination of the item comparisons revealed that the MATADOC ratings differed on three items across the visual and auditory domains, rating the participant’s responsiveness as “MCS” rather than “VS” as rated in the other three measures. In particular, auditory responsiveness was rated higher on the MATADOC (Item 2) during the live condition, albeit at the “inconsistent” response level. Ratings at MCS level for “Awareness of Musical Stimuli” (Item 3) across both live and video conditions support the observations of the child’s responses to his auditory environment. All other items across all four measures rated his responses at VS level.

**Table 2:** Participant 2: demographic information and results from four DOC measures.

Age	8.5 years			
Gender	M			
Diagnosis	Encephalopathy due to bacterial meningitis. Spasticity. Seizure disorder		
Time since injury	>7 years			
Language prior to brain damage	No			
Average session duration	23.25 min			
Inter-rater agreement	78%			
Test–retest agreement	79%			
	**Measures**			
	**MATADOC**	**CRS-R**	**PCC**	**CNC**

Diagnostic outcome	MCS	VS	PVS	Marked coma^∗^
Visual item 1	MCS	VS	VS	VS
Visual item 2	–	–	–	VS
Auditory item 1	MCS	VS	–	VS
Auditory item 2	MCS	–	–	–
Verbal command	VS	VS	VS	VS
Motor	VS	VS	VS	–
Expression	VS	VS	VS	VS

*^∗^Inconsistently responsive to stimulation presented to one sensory modality and not responsive to simple commands. No vocalization*.

Similar to Participant 1, inter-rater agreement was best for items that rated clearly observable behaviors (e.g., Arousal: 100%) and also for items that rated cognitively mediated behaviors that were evidently absent in a patient whose responses are minimally responsive (e.g., Verbal Command: 100%). However, inter-rater agreement was high for a further seven items (at 75% for each item), including three on the Principal Subscale that contribute to higher diagnosis (Visual responsiveness; Auditory responsiveness; Awareness of Musical Stimuli). Overall inter-rater agreement was 78%, with comparable agreement rates between the live condition (81%) and the video condition (79%). Both raters rated higher than the other one at a rate of 16% in the live condition and 9% in the video condition. Although inter-rater agreement was higher for absent responses generally, there was agreement on ratings of responses that were present (i.e., indicative of MCS) in Item 7: Musical responses. These agreed ratings were to behaviors observed relating to musical stimuli categorized as “Timbre” and “Dynamics.” Overall test–retest agreement was the same as for Participant 1 at 79%.

### Case 3

A 15 years-old male had been developing normally prior to acquiring profound anoxic brain damage through asphyxiation, which occurred less than 1 year prior to data collection (see **Table 3**). A diagnostic outcome of MCS was agreed across all four measures. Items within behavioral domains show some differences between different measures.

**Table 3:** Participant 3: demographic information and results from four DOC measures.

Age	15 years			
Gender	M			
Diagnosis	Hypoxic ischemic encephalopathy. Spasticity		
Time since injury	1 year			
Language prior to brain damage	Yes			
Average session duration	23.75 min			
Inter-rater agreement	67%			
Test–retest agreement	73%			
	**Measures**			
	**MATADOC**	**CRS-R**	**PCC**	**CNC**
Diagnostic outcome	MCS	MCS	MCS	Near coma#
Visual item 1	MCS	MCS	VS	VS
Visual item 2	–	–	–	MCS
Auditory item 1	MCS	MCS	–	MCS
Auditory item 2	MCS	–	–	–
Verbal command	VS	VS	VS	MCS
Motor	VS	VS	VS	–
Expression	VS	VS	MCS	NA^∗∗^

*#Consistently responsive to stimulation presented to two sensory modalities and/or partially responsive to simple commands. ^∗∗^Not assessed due to tracheostomy in situ.*

Inter-rater agreement for MATADOC data overall was at 67%. However, differences between raters for the different conditions reflect those seen in Participant 1 to a great degree: the therapist–rater rated higher on twice as many occasions as the observer–rater in both conditions (therapist–rater live: 24%, video: 18%; observer–rater live: 12%, video: 9%). There is some agreement between raters for items that are rated as present/MCS/above “0” across the items rating responsiveness to musical stimuli, (Items 3 and 6, both 87.5%). Of note is the inter-rater agreement (87.5%) for an item rating “Attention to task” where responsiveness is agreed at MCS. There was poorest inter-rater agreement for the item rating responses to Verbal commands. Test–retest agreement was strongest on items rating the auditory domain (item 2) and behavioral responsiveness to musical stimuli (items 3 and 6) notable as responses were rated as present or MCS. Test–retest agreement tended to be strong otherwise only for absent/VS ratings, aside from behavioral responses to “Dynamics” (i.e., a response for a change in the volume of music played) in item 6, where there was 75% test–retest agreement for behaviors that were present. Overall test–retest agreement was at 73%.

### Case 4

A male aged 5.9 years-old had been developing normally prior to acquiring hypoxic ischemic encephalopathy following a cerebral vascular accident that occurred 2.9 years prior to data collection (see **Table 4**). He demonstrated minimal language development at the time of his acquired brain injury. As with participant 3, a diagnostic outcome of MCS was agreed across all four measures, however, items within behavioral domains show widely divergent outcomes across the different measures. MATADOC provided higher ratings than all other measures on two items: Visual responsiveness (Emerging) and Verbal commands (MCS). The CRS-R rated auditory responsiveness lower (VS) than the CNC and MATADOC (MCS). The PCC measure rated motor responses higher (MCS) than the other two measures that rated this domain. Agreement between authors for the MATADOC motor item was difficult to reach as the ratings were highly variable between and within raters, suggesting that the child’s responsiveness bordered somewhere between VS and MCS levels. Inconsistent responses are a typical clinical presentation in DOC populations. We reached consensus on the more conservative rating of “VS” by giving greater weight to inter-rater and test–retest agreement across all possible rating occasions for this item. The participant’s responses can be seen to be highly variable across behavioral domains, spanning VS, MCS, and Emerging.

**Table 4:** Participant 4: demographic information and results from four DOC measures.

Age	5.9 years			
Gender	M			
Diagnosis	Hypoxic ischemic encephalopathy status-post cerebral vascular accident. Spastic quadriparesis		
Time since injury	2.9 years			
Language prior to brain damage	No (minimal)			
Average session duration	19.25 min			
Inter-rater agreement	73%			
Test–retest agreement	80%			
	**Measures**			
	**MATADOC**	**CRS-R**	**PCC**	**CNC**

Diagnostic outcome	MCS	MCS	MCS	Near coma#
Visual item 1	Other (emerging)	MCS	MCS	MCS
Visual item 2	–	–	–	MCS
Auditory item 1	MCS	VS	–	MCS
Auditory item 2	MCS	–	–	–
Verbal command	MCS	VS	VS	VS
Motor	VS	VS	MCS	–
Expression	VS	VS	VS	VS

*#Consistently responsive to stimulation presented to two sensory modalities and/or partially responsive to simple commands.*

Overall inter-rater agreement was 73%. Unlike the other cases presented here, the observer–rater rated much higher than the therapist–rater in both live and video conditions: Live: observer–rater 17% higher compared to therapist–rater rating higher for just 5% occasions; Video: observer–rater rated higher 18% than the therapist–rater who rated higher for just 4% of opportunities. Test–rest agreement was 80% overall. The principal subscale items performed particularly well for both inter-rater and test–retest agreement: Visual responsiveness and arousal: 100%; Auditory responsiveness and Awareness of musical stimuli: 87.5% in both agreements. It is significant that these agreements were reached for behaviors that spanned across VS, MCS, and even Emerging. “Choicemaking” (Item 10) performed well on both inter-rater and test–retest agreement (75% for each). Other items that performed well for test–retest agreement were items rating “Emotional response” (Item 14) and “Choicemaking” (Item 10), both at 75% rating behaviors across VS and MCS. “Musical response” (Item 7) also had high agreement however mostly for absent responses, aside from ratings for “Timbre” which were for both mixed level responses.

### Summary of the Results

Analysis of the MATADOC’s utility, overall and item by item, in relation to three external reference standards and how it was used by two independent raters with children with DOC produced widely varying results. It produced diagnostic outcomes that were in agreement with all other measures in three of the cases. In the fourth, it provided a diagnosis of a higher awareness state than the external reference standards (See **Table 2**). The MATADOC items provided similar ratings for responsiveness as comparable items for the same domains from the other measures (e.g., auditory, visual, verbal command, motor, expression), with three exceptions where it produced ratings of responsiveness higher than that found by the other measures in the visual and auditory domains (see **Tables 2** and **3**). Agreement between MATADOC raters overall ranged from 66 to 73% (mean = 71%) with greater agreement during video ratings than rating of live sessions. In three of the cases, the therapist–rater rated higher than the observer–rater and this occurred to a greater degree during the live condition. Agreement for test–retest was slightly higher ranging from 73 to 80% (mean 78%). Overall, there was greatest agreement where the child’s responsiveness was absent or indicated a VS response although some items performed reasonably well when there were responses over a range of diagnostic categories.

## Discussion

The results are promising for the clinical utility of the MATADOC as a measure for pediatric PDOC. The trends found in the agreement for inter-rater and test–retest ratings reflect those of the larger study to standardize the measure with adults with DOC (Magee et al., 2014). Its performance against external reference standards was also promising, although differences in individual item outcomes require further exploration. It is notable that scores for verbal command items across all measures and cases were overwhelmingly “VS,” with two exceptions that could be anomalies. This highlights the questionable relevance of items that rely on preserved language in pediatric brain-injured patients, and particularly whether language dependent items should contribute to diagnosis in children with PDOC given the issues of language development. Discrepancies between the outcomes of MATADOC items in the visual and auditory domains when compared to other measures (see Cases 2 and 4) are notable. These may be explained as the MATADOC producing false positives, or conversely may suggest that using music as a stimulus may generate greater responsiveness than non-musical stimuli. Higher ratings overall in the auditory domain (item 3) for MATADOC outcomes also occurred in Case 1. However, agreement was not consistent enough between raters or for test–retest ratings to provide an unequivocal score of “MCS.” This picture reflects the inconsistent behavioral patterns typical of a patient with PDOC who might fluctuate between VS and MCS levels of responsiveness, particularly when starting to make functional recovery. The possibility of recovery should not be discounted in this particular case given the patient was within 2 years of injury. A decision to score the patient more conservatively as “VS” on this MATADOC item was made given the inconsistent agreements for MATADOC ratings on this item and the outcome of “VS” on all the comparable items in the auditory domain on the external reference measures. However, the higher level behaviors observed for this item might be explained once more by the use of musical stimuli facilitating greater responsiveness than non-musical stimuli. Clearly further investigation is warranted of item sensitivity in the visual and auditory domains with children, as the MATADOC has been found to have greater sensitivities in the auditory and visual domains than another standardized DOC measure in adults with DOC (O’Kelly and Magee, 2013).

Differences between raters suggest that in three of the cases, the therapist–rater observations were influenced by other factors causing them to rate behaviors at levels indicating greater responsivity. The prevalence of these “over” estimates cannot be explained solely as recall inaccuracies, although it may be that physical proximity afforded the therapist opportunities to see and hear behaviors that were not audible or visible by the observer. This could also explain why both observers tended to rate responses higher within the live condition. The interaction established by a therapist delivering this protocol is a highly intimate one, where the child’s responses are stimulated and then incorporated into musical responses, much as occurs in intimate caregiver–child interactions. Professionals delivering interventions to children who have such profound disabilities invest heavily in tiny responses observed in the child. Providing objective observations for such a subjective experience with a child where treatment teams have so few responses to work with may explain the pattern in therapist ratings for the live condition. This has implications for future testing, where video observations alone may help to enable greater objectivity in ratings. However, until the reasons for discrepancies between ratings in live and video conditions are better understood, further research should continue to consider including both conditions.

### Clinical Utility of the MATADOC Protocol

The current exploratory study has illuminated several logistical considerations for future research and clinical uses of the MATADOC with children. Obtaining optimal levels of arousal for each child was challenging. The children engaged in this study were part of long-term care therapeutic and educational programming, which included participation in sensory-based experiences within an on-site school. Some children were more alert and responsive when seated in their personalized wheelchairs for MATADOC sessions, though this depended upon how long the child had been in his or her chair during educational or therapeutic programming that day, along with other factors that impacted sleep and comfort. Though concerted efforts were made to assure that MATADOC research sessions occurred when the child was in his or her optimal physical positioning, timing of the sessions to promote optimal alertness was difficult to ascertain in advance. Thus, it is recommended that in future research, efforts are made to have daily consultation with the care team to identify the best combination of timing and physical positioning to promote alertness and responsiveness.

Individual children in this study demonstrated unique responses to the music therapist’s voice, sounds delivered via the musical instruments, and to the directives inherent in the assessment sessions. The music therapists engaged in the study had been trained to use music to promote alertness and responsiveness while implementing the protocol; however, some children responded at times to mildly stimulative music and interactions with pacification and sleep. Thus, instead of serving to stimulate arousal, as might be expected due to the acoustic features of the music or the arousing use of the therapist’s voice, the musical stimuli inadvertently served to sedate the child. It would be informative to further explore this relationship to determine if certain children become overstimulated with any form of sensory stimuli, even when experiencing very minimal and progressively presented auditory stimuli.

Some children in the current study appeared to orient and respond best to the novelty of the spoken voice, when the spoken voice contrasted with the musical environment experienced during the implementation of the MATADOC. This finding highlights the alerting capacity of novel sensory stimuli, and the challenge of teasing out which responses are purposeful in nature and which responses are reflexive responses to novel stimuli. Other children alerted to the therapist’s sung voice with guitar accompaniment when they did not alert to isolated musical sounds (such as the playing of a tubular bell as an isolated auditory stimulus). Elements that constitute “stimulative” auditory stimuli may vary from child to child, depending upon his or her history and sensory processing abilities. It is therefore imperative that the MATADOC protocol is presented via live music, so that auditory stimuli and interpersonal interactions may be presented in a way that promotes optimal arousal and purposeful, discriminant responses to stimuli. Such presentation requires a skilled music therapist who is able to provide stimuli in fluid relation to the patient’s unfolding responses.

In order to promote purposeful responsiveness, the MATADOC includes the use of familiar and preferred music. Determining the musical preferences of children with DOC is challenging, especially with those who experienced significant cognitive impairment at birth or early in life. In such cases, it is difficult to determine which music is familiar, salient, and preferred. One must rely on family or caregiver reports or observations of a child’s responses to music, which ultimately may be inaccurate or misinterpreted. Ascertaining musical preference may sometimes rely on the factor of “familiarity,” considering a child’s history of “exposure” to certain music, i.e., television program theme tunes, rather than a direct demonstration of “preference.” During administration of the MATADOC, therapists are encouraged to use a song that is acknowledged as being preferred and familiar to the child. The child’s responsiveness to such a piece of music may fluctuate, and thus, the protocol also allows for therapists to use improvisational exchanges with the child to promote volitional responses.

The current pre-pilot exploratory study has demonstrated the complexity inherent in the assessment of DOC in the pediatric population. Recruitment of the target sample size for this study was challenging due to the frequency of children being diagnosed with cortical visual impairment or not having English as a primary language, an aspect that was tied to the demographic of this urban setting. Following some refinement of the measure, expansion of the research protocol to a multi-site study would provide a larger sample size and enable the testing of reliability and validity of the MATADOC for children and youth.

### Future Directions

There is a demonstrated need for sensory assessment tools that contribute to differential diagnosis of PDOC in pediatric populations with a broad range of etiologies including traumatic and non-traumatic brain injury, perinatal/genetic conditions, and degenerative or neurometabolic disorders (Ashwal, 2003). Since a significant number of children have PDOC as a result of perinatal/genetic conditions (Strauss et al., 2000), a multi-site study with a larger sample size may help distinguish whether the MATADOC is more diagnostically useful for children who have acquired PDOC later in life, versus PDOC from congenital conditions. Considering the potentially high number of children with no language development prior to PDOC, it remains imperative to determine if PDOC can be accurately diagnosed without relying upon language-dependent items. Children who experience severe neurologic injury or dysfunction early in life may demonstrate extensive developmental delays in all areas. Given the complex presentation of such children, it will be important to assess whether the MATADOC reliably discriminates between children who are minimally responsive but conscious versus those who are minimally conscious due to PDOC.

It is possible that additional forms of sensory stimuli not specifically evaluated in the MATADOC may contribute to discriminating purposeful responsiveness in children with PDOC. For example, since children who have acquired PDOC congenitally may have never developed full capacity for sensory integration, the ability to process certain forms of sensory stimuli may be compromised. Some children may demonstrate sensitivities to certain sensory modalities, such as to tactile stimulation, yet may be more tolerant of others, such as vibroacoustic stimuli. Assessor clinicians engaged in the current study anecdotally reported instances of children with PDOC demonstrating increased alertness and responsiveness to vibroacoustic stimuli, delivered via deeply resonant wooden instruments. Such stimulation provides paired auditory and tactile stimulation via low frequency soundwaves, and may promote proprioceptive awareness when placed alongside or beneath the child’s body. Empirical examination of the use of vibroacoustic stimulation for individuals with PDOC is indicated, and is a prerequisite of such stimuli being incorporated into further refinement of the MATADOC protocol as it is tested with a larger cohort.

## Conclusion

The MATADOC provides a sensitive measure of responsiveness to sensory stimuli and early indications suggest that it may contribute to differential diagnosis and enable ongoing evaluation of progress for children with PDOC. Refinement of the measure in alignment with the outcomes of this study, and replication with a larger pediatric sample will strengthen the measure and establish its relevance for this unique population.

## Conflict of Interest Statement

The authors declare that the research was conducted in the absence of any commercial or financial relationships that could be construed as a potential conflict of interest.
